# Association between Lipid Profile, Anthropometric Parameters, and Gestational Diabetes Mellitus: A cross-sectional analysis of NHANES 2015–2023

**DOI:** 10.12669/pjms.42.5.15422

**Published:** 2026-05

**Authors:** Haimeng Luo, Chang Chen, Rongli Xu, Xiuqiong Zheng, Xuechun Wang

**Affiliations:** 1 Haimeng Luo, Department of Obstetrics, Fujian Maternity and Child Health Hospital, College of Clinical Medicine for Obstetrics & Gynecology and Pediatrics, Fujian Medical University, Fuzhou, Fujian Province 350001, P.R. China; 2 Chang Chen, Department of Obstetrics, Fujian Maternity and Child Health Hospital, College of Clinical Medicine for Obstetrics & Gynecology and Pediatrics, Fujian Medical University, Fuzhou, Fujian Province 350001, P.R. China; 3 Rongli Xu, Department of Obstetrics, Fujian Maternity and Child Health Hospital, College of Clinical Medicine for Obstetrics & Gynecology and Pediatrics, Fujian Medical University, Fuzhou, Fujian Province 350001, P.R. China; 4 Xiuqiong Zheng, Department of Obstetrics, Fujian Maternity and Child Health Hospital, College of Clinical Medicine for Obstetrics & Gynecology and Pediatrics, Fujian Medical University, Fuzhou, Fujian Province 350001, P.R. China; 5 Xuechun Wang, Department of Obstetrics, Fujian Maternity and Child Health Hospital, College of Clinical Medicine for Obstetrics & Gynecology and Pediatrics, Fujian Medical University, Fuzhou, Fujian Province 350001, P.R. China

**Keywords:** Anthropometry, Gestational Diabetes Mellitus, Lipids, NHANES

## Abstract

**Background & Objective:**

Gestational diabetes mellitus (GDM) is a common pregnancy complication, affecting approximately 6–9% of pregnancies in United States alone. Women who develop GDM are at higher risk for future Type-II diabetes and cardiovascular disease (CVD). This study examined the association of sociodemographic factors, lipid profiles, and anthropometric measures with GDM history in nationally representative sample.

**Methodology:**

This cross-sectional secondary data analysis used publicly available data from U.S. National Health and Nutrition Examination Survey (NHANES), collected from civilian, non-institutionalized population of United States during 2015–2023. Sample included parous women aged 20–44 years, with GDM status defined by self-report of physician-diagnosed diabetes first occurring during pregnancy. Women with and without history of GDM were compared using survey-weighted statistical tests and multivariable log-binomial regression.

**Results:**

Among 4,504 women analyzed, 8.9% reported history of GDM. Higher education and greater parity, particularly having two or more children, were strongly associated with GDM. Women with GDM had markedly higher prevalence of diabetes. While association between GDM history and abnormal lipid levels was not scientifically significant, obesity and central adiposity were considerably higher in the GDM group, indicating increased metabolic association.

**Conclusions:**

In this nationally representative sample, history of GDM was associated with greater adiposity and higher prevalence of diabetes, while no clear statistically significant association was observed with abnormal lipid levels. These findings suggest that women with prior GDM may represent higher-risk group for future cardiometabolic health problems and may benefit from continued metabolic risk assessment and follow-up after pregnancy.

## INTRODUCTION

Gestational diabetes mellitus (GDM) is one of the most common complications of pregnancy and is defined as glucose intolerance first recognized during pregnancy in women without previously diagnosed diabetes.^1^ With increasing maternal age and obesity, the burden of GDM has risen globally, and national data from the United States also show an increasing prevalence over recent years.^2^,^3^ GDM is clinically important not only because of its implications during pregnancy, but also because it identifies women at elevated risk of subsequent metabolic disease.

A growing body of evidence suggests that women with prior GDM are more likely to develop Type-II diabetes mellitus (T2DM), cardiovascular disease, and other adverse cardiometabolic conditions later in life.^4^ In addition, adiposity and dysregulated lipid metabolism are thought to play an important role in the metabolic profile associated with GDM.^5^ However, population-based evidence describing the relationship of prior GDM with anthropometric measures, lipid abnormalities, and selected sociodemographic factors in nationally representative samples remains limited.

Therefore, this study used data from the National Health and Nutrition Examination Survey (NHANES) 2015–2023 to examine the association of a history of GDM with anthropometric parameters, lipid profile, and relevant sociodemographic characteristics among parous women in the United States. This analysis aimed to provide a clearer population-level picture of the cardiometabolic profile associated with prior GDM and to inform risk stratification and follow-up strategies for women with a history of this condition.

## METHODOLOGY

The present study was cross-sectional secondary analysis of publicly available data from U.S. National Health and Nutrition Examination Survey (NHANES) collected during 2015–2023. NHANES is an ongoing nationally representative survey conducted in United States to assess health and nutritional status of civilian, non-institutionalized population.^6^ It uses complex, multistage probability sampling design and includes household interviews, standardized physical examinations, and laboratory assessments performed in mobile examination centres.^6^ Four NHANES survey cycles (2015–2016, 2017–2018, 2019–2020, and 2021–2023) were pooled for present analysis to obtain adequate sample of women with history of GDM.

### Ethical considerations:

NHANES protocols were reviewed and approved by National Centre for Health Statistics (NCHS) Ethics Review Board, and written informed consent was obtained from all participants by NHANES. For survey cycles included in this analysis, NHANES 2015–2016 was conducted under continuation of Protocol #2011-17; NHANES 2017–2018 under Protocol #2018-01 (effective October 26, 2017), with NHANES 2019–2020 continuing under same protocol; and NHANES 2021–2022 under Protocol #2021-05. As present study used publicly available, de-identified secondary data, additional institutional ethics approval was not required.

### Study population:

The sample was restricted to women of reproductive age (20–44 years) who reported at least one live birth. Women who were currently pregnant at the time of the survey and those with missing data on key variables were excluded. Women with known pre-gestational diabetes (Type-I or Type-II) were also excluded to isolate true GDM cases. After these exclusions, the final analytic sample consisted of 4,504 parous women aged 20–44, of whom 403 had history of GDM and 4,101 did not. All participants provided written informed consent, and the NHANES survey was approved by the National Center for Health Statistics Research Ethics Review Board.

### Sociodemographic and Lifestyle Variables:

Age was recorded in years at time of NHANES interview. Race/ethnicity was self-identified using standard NHANES categories: Non-Hispanic White, Non-Hispanic Black, Hispanic, and Other. Education level was categorized as till grade 9, grade 9 to 11/12, High school, college degree, and college degree and above. Parity was defined as the number of live births reported by the participant and was categorized as one child, two children, and three or more children. Poverty income ratio was calculated and income was categorized as low (<1.3), middle (1.3-<3.5), and high (>3.5). Smoking status was assessed using self-reported NHANES questionnaire data. Smoking status was categorized as active, former, or never smoker. Participants were classified as current/active smokers if they reported currently smoking cigarettes at the time of the survey, as former smokers if they had smoked previously but were not smoking currently, and as never smokers if they reported never smoking cigarettes. Alcohol use was categorized as never alcohol use versus ever alcohol use, based on the corresponding NHANES self-reported alcohol-use variable.

### Anthropometric and blood pressure measurements:

All participants underwent standardized anthropometric measurements by trained health technicians following NHANES protocols. Height was measured to nearest 0.1 cm and weight to nearest 0.1 kg, with participants in light clothing and no shoes. BMI was calculated as weight in kilograms divided by height in meters squared (kg/m²). BMI was treated as continuous variable and categorized (normal <25, overweight 25–29.9, obese ≥30). Waist circumference was measured at the level of the iliac crest to nearest 0.1 cm.^7^ Blood pressure (BP) was measured using an appropriately sized cuff on right arm after five minutes of rest, with average of three consecutive readings used for systolic and diastolic BP. Hypertension was defined as an average systolic BP ≥140 mmHg, diastolic BP ≥90 mmHg, or current use of antihypertensive medication. Sensitivity analyses were done using 2017 ACC/AHA threshold (≥130/80 mmHg).

### Laboratory Measures (Lipid and Glucose Profiles):

Blood samples were collected by venipuncture, and a subsample of participants attended morning session after overnight fast. Serum total cholesterol and HDL cholesterol were measured enzymatically in central laboratory, using CDC-standardized methods. Non-fasting participants only had total and HDL cholesterol.

### Operational definition:

### GDM:

Women were asked if they had ever been told by doctor or health professional that they had diabetes during pregnancy (gestational diabetes). Participants who answered “Yes” were classified as having a history of GDM, provided they did not also report diabetes diagnosed outside of pregnancy.

### Statistical Analysis:

All analyses incorporated complex NHANES survey design, including strata, primary sampling units, and sample weights, to generate nationally representative estimates. As analytic dataset pooled multiple NHANES releases, weighting was handled according to cycle-specific National Center for Health Statistics guidance. Specifically, data from 2015–2016 were combined with NHANES 2017–March 2020 pre-pandemic file and August 2021–August 2023 release using appropriate survey weights for each release, rescaled in proportion to duration represented by each release. Given interruption of data collection during COVID-19 pandemic, analyses combining August 2021–August 2023 with earlier cycles were interpreted cautiously. Participants’ characteristics were compared between patients with and without GDM history using weighted percentages and Chi-square tests for categorical variables. Multivariable survey-weighted regression models were fitted to examine factors associated with history of GDM. Variables considered for multivariable adjustment were selected a priori based on clinical relevance and prior literature and included age, race/ethnicity, education, parity, alcohol use, diabetes status, HDL cholesterol, body mass index, waist circumference, and waist-to-hip ratio. Variables such as poverty income ratio, smoking status, hypertension, and total cholesterol were examined in descriptive analyses but were not retained in final multivariable model because they were either not associated with GDM in bivariate analyses, showed conceptual overlap with other included covariates, or reduced model stability because of sparse data and collinearity. Adjusted risk ratios (aRRs) with 95% confidence intervals (CIs) were reported. Where estimates were based on small cell counts or showed wide confidence intervals, they were considered imprecise and were interpreted as indicating statistical uncertainty rather than strong evidence of association. P-values less than 0.05 were considered significant. Analyses were performed using Stata version 17.0 software (STATACorp, College Station, TX, USA).

## RESULTS

A total of 4,504 women were included in analysis, of whom 403 (8.9%) reported history of GDM. Table-I compares characteristics of women of reproductive age with and without GDM. Educational level showed significant trend (p=0.01), with higher proportion of women with GDM having completed associate degree or higher, indicating increased GDM association with increasing education level (Table-II). Women with GDM were more likely to have higher parity, particularly two or more children. There was a significant association between parity and the GDM in both unadjusted and adjusted models. As shown in Table-II, compared to women with one child, those with two children had nearly twice the association (aRR=2.37; 95%CI: 1.78–3.17), while women with three or more children had even higher association (aRR=2.41; 95%CI: 1.84–3.16). Marital status, income-to-poverty ratio, and place of birth did not show significant associations with GDM.

**Table-I:** Characteristics of the women in reproductive age in NHANES survey with GDM and without GDM, n = 4504.

Characteristics	GDM		No GDM		p-value
	n	(%)	n	(%)	
***Age (years)***					
20-24	78	19.35	640	15.61	<0.001
25-29	153	37.97	1,054	25.70	
30-34	131	32.51	1,277	31.14	
35-44	41	10.17	1,130	27.55	
***Race***					
Mexican American	76	18.86	482	11.75	<0.001
Other Hispanic	37	9.18	413	10.07	
Non-Hispanic White	140	34.74	1,542	37.60	
Non-Hispanic Black	95	23.57	1,033	25.19	
Other race	55	13.65	631	15.39	
***Education***					
Till 9th grade	18	4.47	267	6.51	0.01
Grade 9 to 11/12	49	12.16	450	10.97	
High school	69	17.12	1,017	24.80	
College degree	172	42.68	1,460	35.60	
College grade and above	95	23.57	907	22.12	
***Parity***					
One child	30	7.44	584	14.24	<0.001
Two children	89	22.08	978	23.85	
Three children or more	284	70.47	2,539	61.91	
***Poverty income ratio***					
Low income (<1.3)	143	35.48	1,241	30.26	0.09
Middle income (1.3-<3.5)	149	36.97	1,660	40.48	
High income (>3.5)	111	27.54	1,200	29.26	
***Smoking***					
Current/Active	75	18.61	631	15.39	0.14
Former	70	17.37	831	20.26	
Never	258	64.02	2,639	64.35	
***Alcohol***					
Never alcohol use	340	84.37	3,622	88.32	0.02
Ever alcohol use	63	15.63	479	11.68	
***High blood pressure***					
Yes	168	41.69	1,649	40.21	0.56
No	235	58.31	2,452	59.79	
***Diabetes***					
Yes	134	33.25	524	12.78	<0.001
No	269	66.75	3,577	87.22	
***Total cholesterol***					
<200mg/dl	251	62.28	2,491	60.74	0.55
>=200mg/dl	152	37.72	1,610	39.26	
***HDL Cholesterol***					
>=50mg/dl	212	52.61	2,777	67.72	<0.001
<50mg/dl	191	47.39	1,324	32.28	
***BMI***					
<18.5	3	0.75	58	1.43	<0.001
18.5-24.9	51	12.78	971	23.91	
25-29.9	101	25.31	1,142	28.12	
>30	244	61.15	1,890	46.54	
***Waist Circumference***					
<88cm	54	13.74	1,044	26.46	<0.001
>=88cm	339	86.26	2,902	73.54	
***WHR***					
<0.85	53	13.49	837	21.26	<0.001
>=0.85	340	86.51	3,100	78.74	

*Note:* P-values shown in Table-I represent overall design-based comparisons between women with and without history of GDM for each variable.

**Table-II:** Survey-weighted multivariable regression analysis of factors associated with a history of gestational diabetes mellitus among parous women in NHANES, n = 4504.

Characteristics	Adjusted risk ratio (aRR)	95%CI	p-value
***Age (years)***			
20-24	Reference	-	
25-29	0.89	(0.54, 1.49)	0.44
30-34	0.52	(0.26, 1.04)	0.06
35-44	0.33	(0.11, 0.66)	0.01
***Race***			
Mexican American	Reference	-	
Other Hispanic	0.58	(0.30, 1.12)	0.07
Non-Hispanic White	0.86	(0.59, 1.24)	0.22
Non-Hispanic Black	0.52	(0.36, 0.75)	0.02
Other race	0.74	(0.36, 1.52)	0.21
***Education***			
Till 9th grade	Reference	-	
Grade 9 to 11/12	2.34	(1.57, 3.64)	0.001
High school	1.46	(0.53, 3.99)	0.25
College degree	2.37	(1.30, 3.44)	<0.001
College grade and above	3.37	(1.96, 4.85)	0.001
***Parity***			
One child	Reference	-	-
Two children	2.37	(1.78, 3.17)	0.01
Three children or more	2.41	(1.84, 3.16)	0.01
***Alcohol***			
Never alcohol use	Reference	-	-
Ever alcohol use	2.36	(0.75, 4.44)	0.09
***Diabetes***			
Yes	3.10	(0.76, 5.65)	0.07
No	Reference	-	–
***HDL-Cholesterol***			
>=50mg/dl	Reference	-	
<50mg/dl	1.20	(0.86, 1.67)	0.14
***BMI***			
<18.5	Reference	-	–
18.5-24.9	1.71	(1.16, 2.48)	0.001
25-29.9	1.75	(0.92, 3.34)	0.07
>30	1.99	(1.28, 3.09)	0.02
***Waist Circumference***			
<88cm	Reference	-	–
>=88cm	1.24	(0.38, 4.08)	0.52
***WHR***			
<0.85	Reference	-	
>=0.85	1.58	(0.67, 2.50)	0.49

aRR = adjusted risk ratio; CI = confidence interval. Reference categories are shown explicitly for each variable. Variables such as poverty income ratio, smoking, high blood pressure, total cholesterol were assessed in descriptive analyses but were not retained in the final adjusted model because of lack of contribution to model fit, overlap with related covariates, or instability of estimates. Estimates with wide confidence intervals should be interpreted cautiously.

Alcohol use status differed significantly between women with and without a history of GDM in the unadjusted analysis (Table-I). However, the proportion reporting no alcohol use was lower among women with GDM than among those without GDM (84.4% vs. 88.3%), and this association did not remain statistically significant after multivariable adjustment (Table-II). Prevalence of hypertension and smoking status did not significantly differ between two groups. There was over 3-fold higher association of having diabetes in women with GDM (aRR=3.10; 95%CI: 0.76–5.65). In terms of anthropometric indicators, obese women had nearly double the association of GDM compared to those with normal BMI (aRR=1.99; 95%CI: 1.28–3.09).

## DISCUSSION

The results demonstrated that approximately 8.9% of parous women in NHANES 2015–2023 dataset reported history of GDM, underscoring relatively high prevalence of this condition in general population.^8^ This prevalence, while within range of previous estimates, reinforces notion that GDM is common complication during pregnancy that warrants sustained clinical attention even after delivery. Our observation that older maternal age correlates with an increased association of GDM resonates with earlier research that has repeatedly underscored role of age-related metabolic decline in pathogenesis of GDM.^9^

In addition to age, study highlights significant racial and ethnic disparities in prevalence of GDM. Non-Hispanic Black women had lower prevalence and higher association with GDM. Previous studies also confirmed that there are certain racial and ethnic groups that face greater burden of metabolic diseases, due to combination of genetic predispositions and socio-environmental factors.^10^

Women with higher parity especially those with more than or equal to two children, showed significantly higher odds of GDM compared to primiparous women. This observation strongly supports hypothesis that repeated pregnancies contribute to cumulative metabolic stress, which in turn may predispose women to disturbances in glucose regulation.^11^ Metabolic toll of multiple gestations could be associated with long-term changes in insulin sensitivity and beta-cell function, thereby increasing likelihood of persistent metabolic abnormalities.^12^

Smoking and alcohol consumption in this study did not differ significantly between women with and without GDM, indicating that these factors might not be critical in the immediate pathogenesis of GDM, and offering interesting area for comparison with previous literature.^13^ In contrast, there was strong association between GDM and obesity and central adiposity. Women with a history of GDM exhibited notably higher body mass index (BMI) and larger waist circumferences compared to those without GDM. These anthropometric indicators are proxies for overall adiposity and central fat distribution, which are well-known contributors to insulin resistance and metabolic dysregulation.^14^

These findings may also have relevance for regional contexts such as Pakistan, where burden of maternal metabolic disorders is increasing and postpartum follow-up may be inconsistent across settings. Although present analysis was based on U.S. NHANES data, observed clustering of prior GDM with greater adiposity and higher diabetes prevalence highlights importance of recognizing GDM as an early marker of future cardiometabolic vulnerability. In Pakistan, these findings may support need for strengthened screening during pregnancy, improved postpartum monitoring of women with prior GDM, and integration of lifestyle counselling into maternal and primary care services.

### Limitations:

First, its cross-sectional design affects causal or temporal relationships between GDM and cardiometabolic outcomes. Second, GDM status has been self-reported by study participants, which might introduce possibility of misclassification and recall bias. Finally, NHANES data lacks information on GDM diagnosis timing or number of years since index pregnancy, limiting their ability to assess long-term risk trajectories.

## CONCLUSION

The study shows that history of GDM is linked to heightened association for long-term metabolic complications. Older age, increased parity, and greater adiposity were closely associated with GDM and suggest an higher association with chronic conditions such as Type-II diabetes and cardiovascular disease. These findings advocate for early identification, sustained lifestyle modifications, and continuous clinical monitoring in affected women.

### Recommendations:

Future research should use prospective longitudinal designs to clarify the temporal relationship between GDM and later cardiometabolic abnormalities and to identify factors that mediate long-term risk.

### Author’s contributions:

**HL and CC:** Literature search, study design and manuscript writing.

**RX, XZ and XW:** data collection, data analysis and interpretation. Critical Review.

**XW:** Manuscript revision and validation and is responsible for the integrity of the study.

All authors have read and approved the final manuscript.
